# The relationship of human tissue microRNAs with those from body fluids

**DOI:** 10.1038/s41598-020-62534-6

**Published:** 2020-03-27

**Authors:** Chunmei Cui, Qinghua Cui

**Affiliations:** 1 Department of Biomedical Informatics, Department of Physiology and Pathophysiology, Center for Noncoding RNA Medicine, MOE Key Lab of Cardiovascular Sciences, School of Basic Medical Sciences, Peking University, 38 Xueyuan Rd, Beijing, 100191 China; 2 Center of Bioinformatics, Key Laboratory for Neuro-Information of Ministry of Education, School of Life Science and Technology, University of Electronic Science and Technology of China, Chengdu, 610054 China

**Keywords:** Bioinformatics, Computational biology and bioinformatics

## Abstract

It is known that many microRNAs (miRNAs) stably exist in various body fluids, however, the relationship of body fluids miRNAs (BF-miRNAs) with those from tissues (T-miRNAs) remains largely unclear but is important for understanding the potential of BF-miRNAs to be biomarkers of specific diseases. Here by analyzing miRNA expression data from 40 human healthy tissues and those from human body fluids, including plasma, serum, urine, bile, and feces, we revealed a positive correlation between BF-miRNAs and T-miRNAs. Moreover, plasma and serum have the most communication with pericardium, adipose, liver, and spleen. Urinary miRNAs show the highest correlation with kidney miRNAs. For fecal miRNAs, gastrointestinal tract (colon, ileum, jejunum, small intestine, stomach, proximal colon, duodenum, and distal colon) miRNAs show the strongest correlation. Moreover, miRNA set enrichment analysis revealed that highly expressed fecal miRNAs are mostly associated with gastric and colon cancers etc. Additionally, bile miRNAs from suspected cholangiocarcinoma patients show a positive correlation with the cholangiocarcinoma tumor tissue. Interestingly, the relationship of BF-miRNAs and T-miRNAs shows significant sex differences. Serum miRNAs showed higher correlation with T-miRNAs in males, whereas plasma miRNAs and urine miRNAs showed higher correlations with T-miRNAs in females. These findings together indicate a potential role of BF-miRNAs as biomarkers to monitor corresponding specific human diseases.

## Introduction

MicroRNAs (miRNAs) are one class of important small non-coding RNA molecules, which mainly negatively regulate gene expression at the post-transcriptional level with important roles in a variety of key biological processes^1^. Therefore, the dysfunctions of miRNAs could be associated with a wide range of diseases^2^, including cancer and cardiovascular disease. It is well known that miRNAs can be found in various human tissues^3^. Moreover, the existence of blood miRNAs suggests their potential to be biomarkers^4^. Currently, there have been numerous studies focusing on miRNAs in body fluids as biomarkers in certain diseases^5–7^, such as cancers, immune-related diseases. We previously revealed the relationship between whole-blood miRNAs and tissue miRNAs^8^, suggesting potential cancer defensive roles of blood miRNAs. Interestingly, comparing with venous plasma miRNAs, arterial plasma miRNAs showed stronger correlations with miRNAs from various tissues in rats^9^. More recently, the stable presence of miRNAs has been found in multiple body fluids of healthy people and patients^10,11^, including plasma, serum, urine, bile, and feces. Systematically exploring the relationship between body fluid miRNAs (BF-miRNAs) with those in various tissues (T-miRNAs) will be of great help in understanding the roles of BF-miRNAs in human health and diseases.

In this study, we performed a bioinformatics analysis for miRNA expression profiles from human body fluids and various tissues. As a result, we found a global tendency of significantly positive correlation between BF-miRNAs and T-miRNAs in healthy people. Similarly, in cholangiocarcinoma patients, there was a highly positive correlation between bile miRNAs and miRNAs from tumor tissue. Furthermore, we observed the most communication tissues of each body fluid. To be specific, plasma and serum miRNAs with those in pericardium, adipose, and liver, urinary miRNAs with kidney miRNAs, fecal miRNAs with those from gastrointestinal tract (colon, ileum, jejunum, small intestine, stomach, proximal colon, duodenum, and distal colon) show the most correlations. Finally, sex differences in the relationship between BF-miRNAs and T-miRNAs were observed.

## Materials and methods

### miRNA expression datasets

We obtained the miRNA expression dataset of 40 healthy human tissues from Liang *et al*.’s study^3^. The miRNA expression dataset of human body fluids which includes plasma, serum, urine and bile were obtained from Sinivasan *et al*.’s study^11^ and the miRNA expression dataset of feces were obtained from Liu *et al*.’s study^10^. Besides, we downloaded miRNA expression dataset of cholangiocarcinoma (CHOL) from the TCGA database^12^.

### Data analysis

For all expression data, we deleted the NA values and averaged the expression values of the redundant IDs. Then we mapped the expression profile of tissue miRNAs and each body fluid miRNAs according to the miRNA name. Spearman’s correlation analysis was performed using R, a free statistical platform (https://cran.r-project.org/). To compare the relationship of BF-miRNAs and T-miRNAs between male and female, we separated the samples into two groups using gender information of samples. miRNA set enrichment analysis was performed using the TAM tool v2.0^13,14^.

## Results

### BF-miRNAs show globally positive correlations with tissue miRNAs in healthy individuals

**Figure 1:**
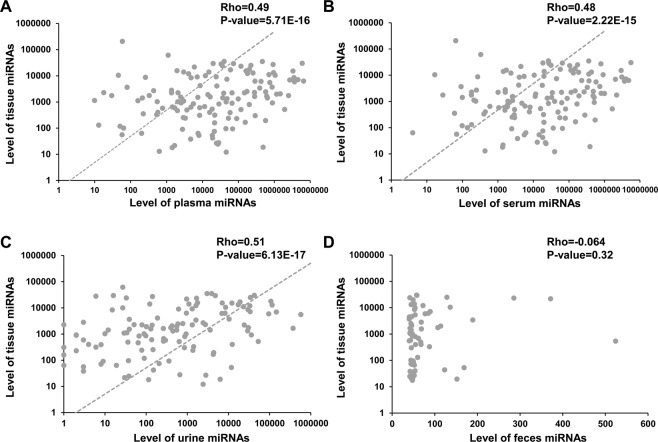
The whole relationship between body fluid miRNAs and tissue miRNAs. (**A**) Correlation of plasma miRNAs and tissue miRNAs. (**B**) Correlation of serum miRNAs and tissue miRNAs. (**C**) Correlation of urine miRNAs and tissue miRNAs. (**D**) Correlation of fecal miRNAs and tissue miRNAs.

**Figure 2:**
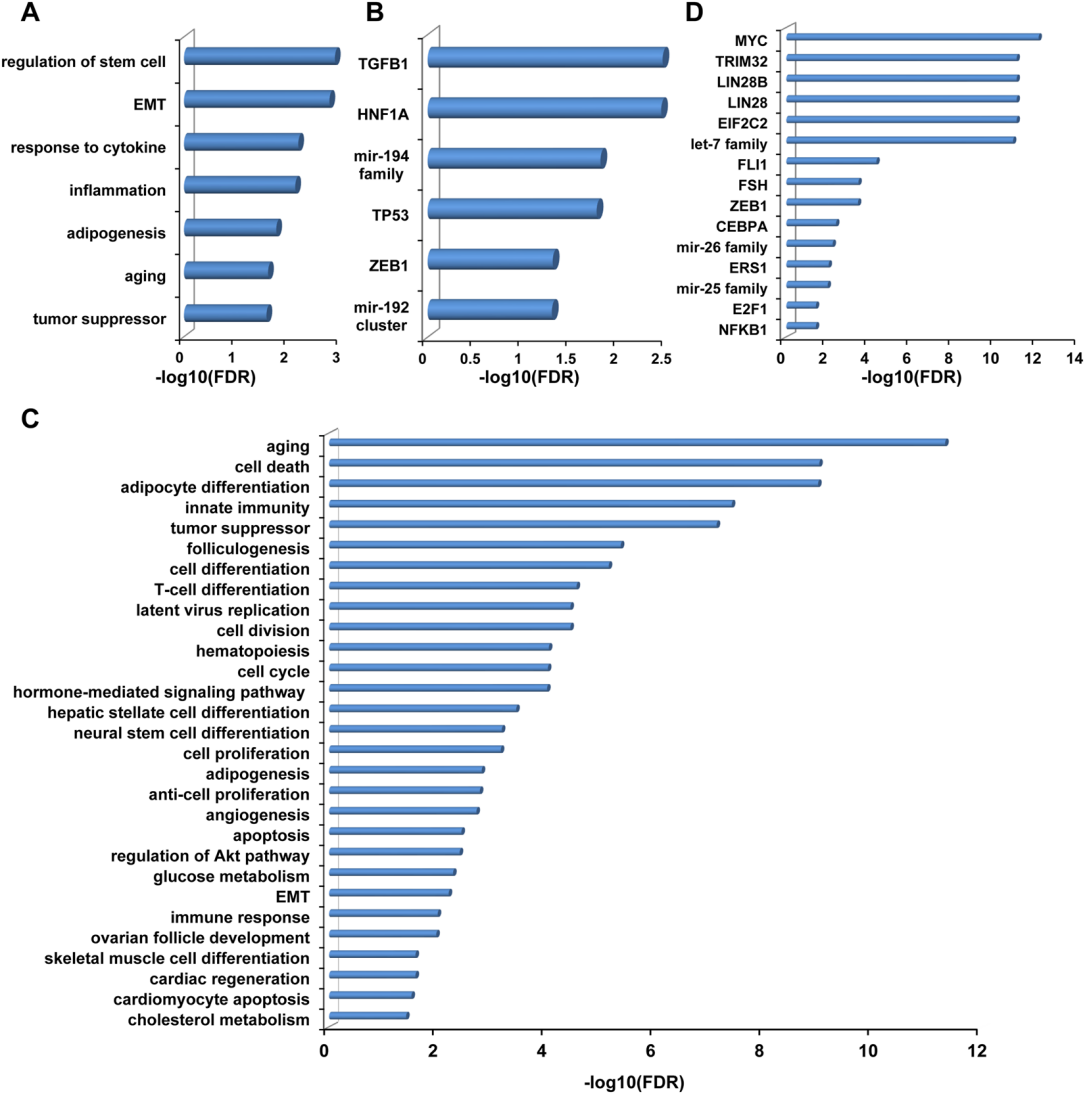
The miRNA set enrichment analysis of highly expressed body fluid miRNAs. (**A**) Results of functional enrichment analysis for highly expressed miRNAs in feces. (**B**) Other enrichment analysis results of highly expressed fecal miRNAs, including miRNA cluster, family, and transcription factors (TFs). (**C**) Results of functional enrichment analysis for highly expressed miRNAs in plasma, serum and urine. (**D**) Other enrichment analysis results of highly expressed miRNAs in 3 body fluids.

As a result, we observed a globally significant positive correlation between human tissue miRNAs and three types of BF-miRNAs, including plasma (rho = 0.49, P-value = 5.71E-16, Spearman correlation; Fig. 1A), serum (rho = 0.48, P-value = 2.22E-15, Spearman correlation; Fig. 1B), urine (rho = 0.51, P-value = 6.13E-17, Spearman correlation; Fig. 1C). However, we failed to observed a correlation between fecal miRNAs and T-miRNAs (rho = −0.064, P-value = 0.32, Spearman correlation; Fig. 1D), which might be due to the lower substance exchange of feces with other tissues except for gastrointestinal tissues in comparison to other three body fluids. Then we selected feces highly expressed miRNAs with expression level >100 and performed enrichment analysis using TAM. As a result, these miRNAs are mainly involved in the following functions (FDR <= 0.05, Table S1), regulation of stem cell, EMT, response to cytokine, inflammation, adipogenesis, aging, and tumor suppressor (Fig. 2A). In addition, these miRNAs are enriched in mir-192 cluster and mir-194 family (Fig. 2B). Moreover, these miRNAs are mainly regulated by four transcription factors (TFs), TGFB1, HNF1A, TP53, and ZEB1 (Fig. 2B). For disease term, these miRNAs are enriched in 38 diseases, among which colon cancer is the top No.2 disease (FDR = 1.0E-3). Besides, stomach cancer is also significant (FDR = 0.0176).

The global relationship between BF-miRNAs and T-miRNAs has been dissected. It is necessary to know the functional roles of the highly expressed miRNAs in the body fluids. We have uncovered the enriched functions, clusters, families, and TFs of the highly expressed miRNAs in feces using the TAM tool. Here, using the same procedure, we firstly selected highly expressed miRNAs in plasma (expression level >10000), serum (expression level >10000) and urine (expression level >100). We found those miRNAs are highly overlapped in 3 body fluids. For those highly expressed miRNAs in three types of body fluids, serum miRNAs are all present in plasma, 74% (14/19) of serum miRNAs are in urine, and 75% (18/24) of plasma miRNAs are in the urine. Therefore, we performed enrichment analysis for those miRNAs which was highly expressed in all three body fluids. As a result, the top terms of significantly enriched function include aging, cell death, adipocyte differentiation, innate immunity, and tumor suppressor etc (Fig. 2C). The terms were listed in Table S3. Also, those miRNAs mainly enrich in the let-7 family, mir-26 family, mir-25 family and are significantly regulated by the TFs, for instance, MYC, TRIM32, LIN28B and so on (Fig. 2D). Besides, we observed vascular hypertrophy and lymphoma are the most significant related diseases.

### BF-miRNAs showed biased correlations with miRNAs in specific tissues

**Figure 3:**
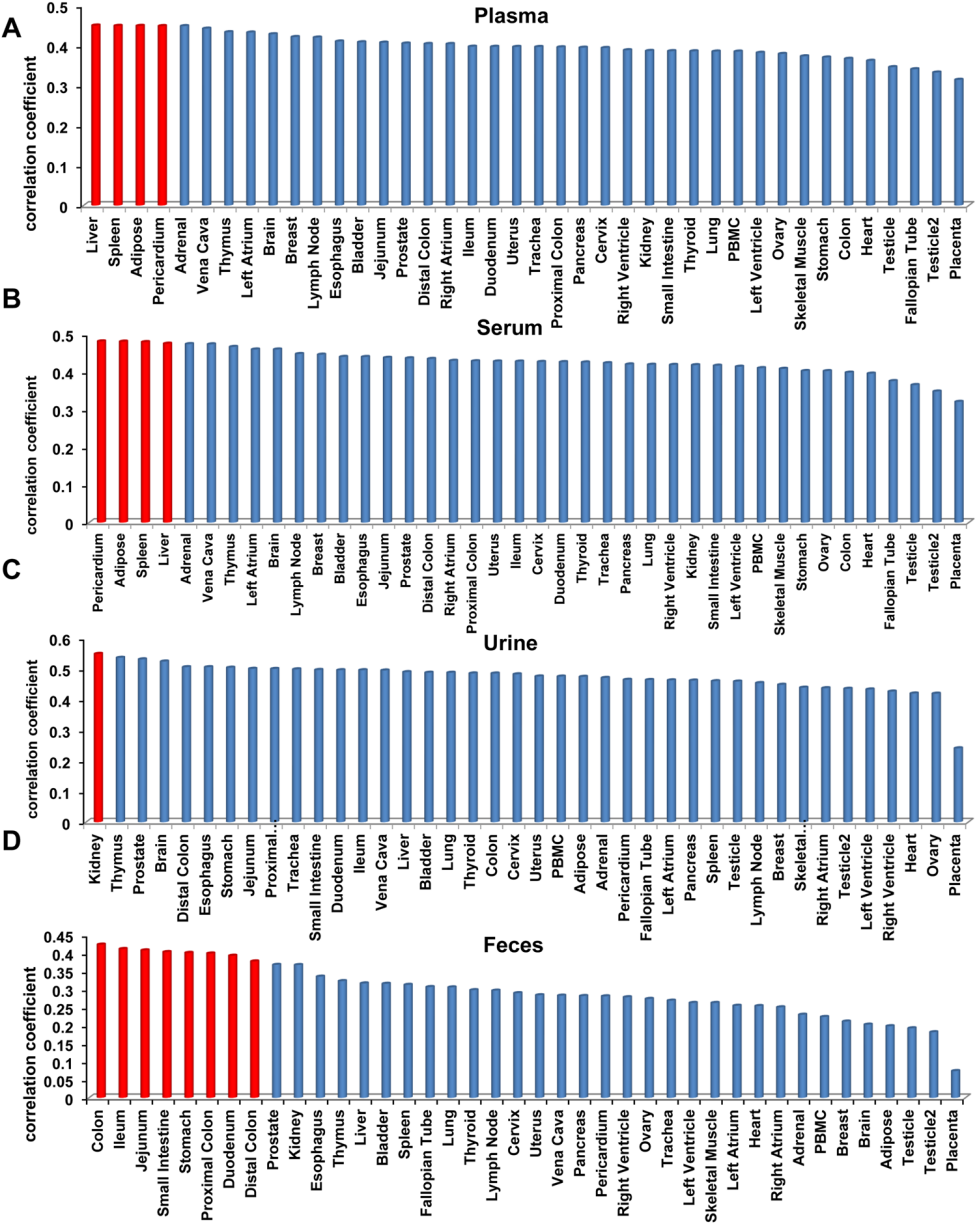
Analysis the relationship of body fluid miRNAs with miRNAs in 40 tissues. (**A**) Correlations between plasma miRNAs with those in the 40 tissues. (**B**) Correlations between serum miRNAs with those in the 40 tissues. (**C**) Correlations between urine miRNAs with those in the 40 tissues. (**D**) Correlations between feces miRNAs with those in the 40 tissues.

We further studied the relationship of miRNA expression between the four body fluids and the 40 healthy tissues. Plasma and serum show highly similar tendency of correlation with the 40 tissues (Fig. 3A,B). Pericardium, adipose, liver and spleen are the top tissues of strongest correlation with both plasma and serum. It is well known that pericardium, liver, and spleen are indeed organs with a profusion of blood supplies^15–17^. For adipose, a previous study has proposed the adipose cells can release large amounts of miRNAs into the plasma^18^, suggesting that plasma and serum miRNA biomarkers could do better on diseases related with heart, liver, spleen, and adipose than on other diseases. Interestingly, urinary miRNAs have the highest correlation with kidney-borne miRNAs (rho = 0.55, P = 6.74E-22, Spearman correlation; Fig. 3C), which hints the prospect of urinary miRNA signature in kidney-related diseases. In addition, several studies have reported urinary miRNAs as biomarkers for the diagnosis of kidney disease^19,20^. As mentioned above, feces should have direct contact with gastrointestinal tissues. As a result, we found that the miRNAs from colon, ileum, jejunum, small intestine, stomach, proximal colon, duodenum, and distal colon which all belong to gastrointestinal tract, show the strongest correlations with fecal miRNAs (Fig. 3D). This result means that fecal miRNAs could mostly communicate with gastrointestinal tract and thus could monitor the physiological and even pathophysiological status of gastrointestinal tract. In short, the miRNA expression status of not only the above four body fluids but also all body fluids might be the same as their most frequently communicated tissues. Taking account of the characteristics of miRNA expression, we also applied other proper correlation methods, including Kendall coefficient of concordance and distance correlation. The results showed high similarity with that of spearman correlation. For example, liver and pericardium are still the tissues of more correlation for serum and plasma. For urinary and fecal miRNAs, the most correlation tissues are also similar (Table S3), which further suggested the stable existence of contacts between body fluids and specific tissues.

### Bile miRNAs show positive correlation with cholangiocarcinoma tumor tissue

**Figure 4:**
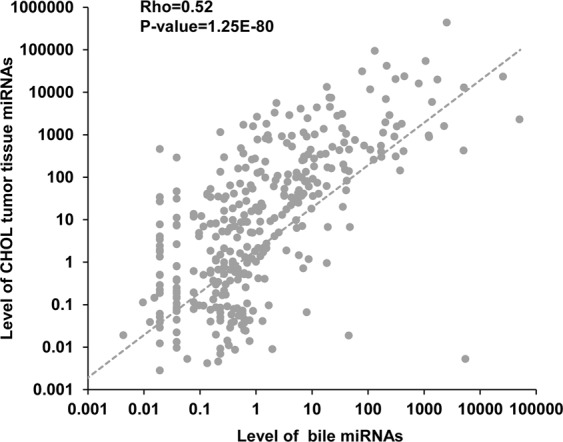
The relationship of bile miRNAs with tumor tissue miRNAs in cholangiocarcinoma patients.

Circulating miRNAs only partially originate from the secretion of tumor tissue and may also come from sources such as tissue damage and micrometastatic cells^21^. We have found a significantly positive correlation between the miRNA expression of tissues and body fluids in normal samples, however, the overall relationship of miRNA expression between pathological tissues and body fluids is not known. Therefore, we analyzed the bile miRNAs from suspected CHOL patients and the miRNAs from CHOL tumor samples in TCGA database. As shown in Fig. 4, there is a significantly positive correlation between bile-borne miRNAs and the miRNAs from tumor tissue (rho = 0.52, P = 1.25E-80, Spearman correlation), suggesting that T-miRNAs have similar expression state with BF-miRNAs in the abnormal condition. The results hint the body fluid miRNAs might be the ideal biomarkers for diagnosis, development, and prognosis of the diseases.

### Comparison of the relationship of BF-miRNAs and T-miRNAs between male and female

**Figure 5:**
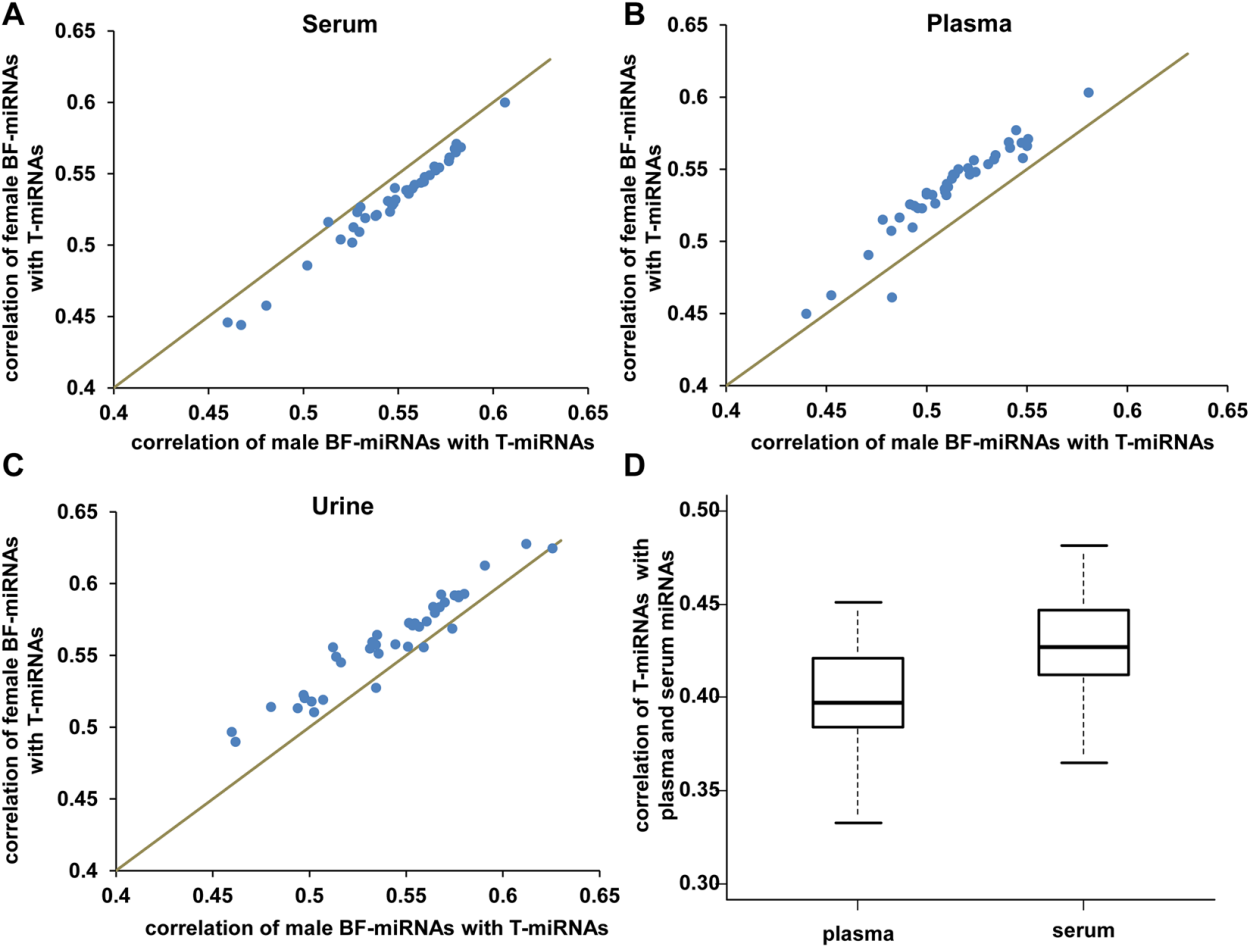
Analysis of sex differences in the relationship of tissue miRNAs and body fluid miRNAs. The correlation coefficients of tissue miRNAs with male and female miRNAs from serum (**A**), plasma (**B**) and urine (**C**) are plotted. The line represents the correlation coefficients of body fluid miRNAs with tissue miRNAs between males and females are equal. (**D**) Comparison of the correlation of tissue miRNAs with plasma and serum miRNAs.

We previously have reported sex-biased miRNAs in healthy human tissue and peripheral blood cells and revealed some patterns that female-biased miRNAs are more conserved and are significantly associated with metabolism process and cell cycle process^22^. Therefore, we studied whether there is sex difference between the relationship of BF-miRNAs and T-miRNAs. We separated the miRNA expression profiles of body fluids by the gender information of the samples and compared the correlation of BF-miRNAs and T-miRNAs between males and females. Consequently, It was indeed found that the serum miRNAs had a significantly higher correlation with T-miRNAs of 40 tissues in males (paired t-test P-value = 1.90E-20, Fig. 5A), whereas miRNAs from female samples had significantly higher correlation with T-miRNAs in plasma (paired t-test P = 3.25E-18, Fig. 5B) and urine (paired t-test P-value = 4.32E-12, Fig. 5C). In addition, we also compared the correlation of T-miRNAs with plasma and serum miRNAs. We found the correlations of serum with tissues are significant higher than that of plasma with tissues (median: 0.43 vs. 0.40, P-value=2.73E-23, Fig. 5D), which suggested serum miRNAs could perform better to reflect the state of tissues.

## Discussions

In conclusion, we revealed that miRNAs of body fluids tend to be positively correlated with various human tissues globally. For plasma and serum miRNAs, they show the strongest correlations with pericardium, adipose, liver, and spleen miRNAs. Urinary miRNAs have the highest correlation with kidney miRNAs. And fecal miRNAs show the strongest correlations with miRNAs from colon, ileum, jejunum, small intestine, stomach, proximal colon, duodenum, and distal colon. In addition, we uncovered that feces highly expressed miRNAs are mostly enriched in functions regulation of stem cell, EMT, response to cytokine, inflammation, adipogenesis, aging, and tumor suppressor, and diseases of digestive system cancer (colon cancer, stomach cancer etc). Similarly, we found the most expressed miRNAs in all other 3 body fluids. Those miRNAs are enriched in the functions of aging, cell death, adipocyte differentiation, innate immunity, and tumor suppressor. Furthermore, bile miRNAs have a significantly positive correlation with miRNAs in tumor tissue under disease status. Intriguingly, in different types of body fluids, the relationship of BF-miRNAs and T-miRNAs shows different sex-preferences. Specifically, male BF-miRNAs are significantly higher correlated with T-miRNAs in serum, whereas the opposite pattern appears in plasma and urine. This finding suggests that for blood-borne miRNA based disease diagnosis, it could be better to take serum samples for male patients and plasma for female patients. Together, these findings suggest frequent communication between body fluids and corresponding tissues and potential miRNA signature in body fluids, which are not random but have regular patterns. And thus body fluid miRNAs could be a non-invasive biomarker for facilitating the diagnosis and prognosis of various human diseases. Nevertheless, it should be noted that the answers to a number of important questions are still unknown. For example, where the body fluid miRNAs are from and what their functions are. Therefore, more analysis is needed when more data becomes available in the future.

## Supplementary information

Supplementary table S1.

Supplementary table S2.

Supplementary table S3.

Supplementary information.

## Data Availability

The raw data used in our study are from the publications^3,10,11^.
