# Supplementary material for: The relationship of human tissue microRNAs with those from body fluids
Source: Sci Rep. 2020 Mar 27;10:5644. doi: 10.1038/s41598-020-62534-6 (PMC7101318; doi:10.1038/s41598-020-62534-6)
Supplement: Supplementary file 4 — Supplementary information. [file 41598_2020_62534_MOESM4_ESM.docx]

Supplementary material

Table S1 The enriched terms of highly expressed miRNAs in feces

Table S2 The enriched terms of highly expressed miRNAs in the three body fluids

Table S3 The correlation between BF-miRNAs and T-miRNAs with other correlation methods
